# Etiological profile of peripheral neuropathies in an academic hospital in southern Morocco

**DOI:** 10.1186/s41983-022-00531-4

**Published:** 2022-08-20

**Authors:** Anselm Poda, Raymond Klevor, Aouatif Salym, Imad Sarih, Sami Salhi, Louhab Nissrine, Najib Kissani

**Affiliations:** 1 Neurology Department, Mohammed VI University Medical Center of Marrakesh, P. O. Box 2360, Marrakesh, Morocco; 2 Neuroscience Research Laboratory, Faculty of Medicine and Pharmacy of Marrakesh, Cadi Ayyad University, P. O. Box 7010, Marrakesh, Morocco

**Keywords:** Peripheral neuropathy, Etiology, Diabetes, Acute polyradiculoneuropathy, Alcohol, Morocco

## Abstract

**Background:**

Peripheral neuropathies constitute a common complaint in general and neurology practice, and are a source of handicap to patients. Epidemiological data in the Middle East and North Africa region as well as in the African continent are sparse. Nevertheless, regional etiological profiles are crucial in navigating the diagnostic maze of neuropathies. This study outlines the etiological profile of peripheral neuropathies in an academic hospital in southern Morocco.

**Results:**

A total of 180 cases were recorded in a span of 8 years (22.5 cases per year). The mean age of patients was 42.35 years. Male gender was predominant (68.88%), with a sex ratio of 2.2. Motor symptoms were the most frequently reported (86.6%). The axonal form (40.56%) was the most frequently encountered electrophysiologic form. The most frequent etiologies in the study were diabetes (26.7%), acute polyradiculoneuropathy (26.1%) and amyotrophic lateral sclerosis (16.1%). Alcohol neuropathy was found in 2.2% of the cohort. No cause was found in 5% of cases. Outcome was mostly favorable under treatment, although 10 deaths due to acute polyradiculoneuropathy were recorded (mortality = 21.3%).

**Conclusions:**

Knowledge of the etiological profile of peripheral neuropathies should guide clinicians to an early diagnosis and aid in an adapted management of patients.

**Supplementary Information:**

The online version contains supplementary material available at 10.1186/s41983-022-00531-4.

## Background

Peripheral neuropathies are among the commonest complaints in clinical practice. It is a diagnosis the clinician must be familiar with. Etiological labeling can, however, be a diagnostic nightmare. The reason is probably the varied presentations and myriad etiologies [1].

The need to make the right diagnosis is seen in the fact that some peripheral neuropathies could be life-threatening as is the case of acute polyradiculoneuropathies [2]. The occurrence of neuropathies in various systemic conditions where they could represent modes of revelation of these conditions makes it important to recognize them in the clinical setting. In addition, neuropathies secondary to a specific condition might benefit from management of etiology rather than mere symptomatic treatment [3, 4].

Importantly, deciphering the causes of peripheral neuropathies is helped by a sound knowledge of regional profiles. Besides the insufficiency of data on the incidence of polyneuropathies in general as pointed out by Visser and colleagues, there is an unfortunately marked paucity of research on the subject in Morocco [5, 6]. This work cumulates data on peripheral neuropathies in an academic hospital over the period of 8 years so as to decipher etiological profiles of these conditions in the country, which could inform later diagnostic approaches.

## Methods

The present study is a retrospective, descriptive study based on patient data obtained from the archives of the Neurology Department at the Mohammed VI University Medical Center in Marrakesh, from January 2002 to December 2009.

Files selected had to have the diagnosis of peripheral neuropathy, be discussed during staff meetings, and contain complete data on patient profile, symptom presentation and laboratory and electrophysiological workup. Neuropathies that were traumatic in origin or entailed conflict mechanisms such as carpal tunnel syndrome were excluded from the study. In addition, cranial pair neuropathy was not included in the present study.

Out of a total of 720 patients hospitalized within the 8-year period of the study for peripheral neuropathies, only 180 patient files met the criteria.

For each patient file, clinical data were obtained and included demographic characteristics and clinical presentation. Patient history included medical conditions the patient was followed-up for, as well as the existence of drug use, both prescription and non-prescription. Patients with a history of cancer, tuberculosis or auto-immune disorder or other pathologies had to indicate the agents used and the existence of symptoms before or after drug initiation.

Also, data on laboratory findings, electrophysiological studies and imaging studies were detailed. In addition, etiological diagnosis was noted each time this was made (see Table 1 for definition of etiologies).

**Table 1:** Criteria for making etiological diagnosis of peripheral neuropathies [7]

Diagnosis	Definition of neuropathy
Diabetes	Patient with diabetes mellitus presenting peripheral neuropathy in whom workup finds no alternative cause
Acute polyradiculoneuropathy	Acute (< 4 weeks) of ascending bilateral symmetric peripheral neuropathy. Deep tendon reflexes are abolished or diminished; with prolonged distal latencies and F waves, and reduced motor conduction velocities
Motor neuron disease	Association of upper and lower motor neuron impairment in diffuse distribution with fasciculations; neuronopathy on electrophysiological evaluation; absence of compressive or other secondary cause
Immunologic	Peripheral neuropathy in the context of (either inaugurating or associated with) an autoimmune condition such as Lupus, sarcoidosis, Sjogren’s…
Idiopathic	Peripheral neuropathy with negative paraclinical findings despite extensive workup
Medication-induced	Peripheral neuropathy in the context of exposure to a medication known to cause neuropathy. Neuropathy is either inaugural or an underlying neuropathy is worsened when medication is started. Typically patients are undergoing anti-cancer medications such vincristine, cisplatine, nucleosidic analogue anti-retroviral agents, dapsone and phenytoin. Etiologic workup is unremarkable, and patients tend to stabilize or recover upon medication retraction
Infective	Neuropathy in the context of infection diagnosed on cerebrospinal fluid (CSF) analysis. CSF evaluation includes cell count, protein, glucose levels, soluble antigens, culture, and polymerase chain reaction (PCR)
Friedreich ataxia	Typical sensory peripheral neuropathy in a patient diagnosed with Friedreich ataxia
Alcohol neuropathy	Typical sensory neuropathy in patients who consume alcohol. Patients describe painful (burning) sensations in limbs
Paraneoplastic	Subacute peripheral neuropathy in patients with neoplasm. The neuropathy could be the initial complaint for which the workup reveals the neoplasm. Positivity of onconeuronal antibodies, the finding of a tumor, and recuperation after removal of tumor align with the diagnosis
Critical illness polyneuropathy	Polyneuropathy in patients with prolonged stay in intensive care unit. Typically, patients would have received neurotropic drugs such as curare. Workup is usually unremarkable or could reveal metabolic anomalies
Chronic polyradiculoneuropathy	A Guillain–Barré-type presentation with chronic onset. Patients report tingling in extremities and then motor impairment in a length-dependent fashion
Deficiency neuropathy	Peripheral neuropathy in the context of vitamin deficiency. Patients present marked sensory signs with tingling and loss of proprioception. Vitamin B 12 deficiency might be associated with upper motor impairment (subacute combined degeneration of spinal cord)
Amyloid	Peripheral neuropathy in the context of amyloidosis, primary or secondary. Biopsy is required to show infiltration by the abnormal protein
Toxic	Peripheral neuropathy in a patient with known exposure to a toxin with potential to cause neuropathy. After excluding other etiologies, toxic neuropathy is retained. Patients are typically workers in manufacturing plants, painters, farmers, to name a few. Patients may also be drug-addicts (example: paint-thinner sniffers)

**Fig. 1:**
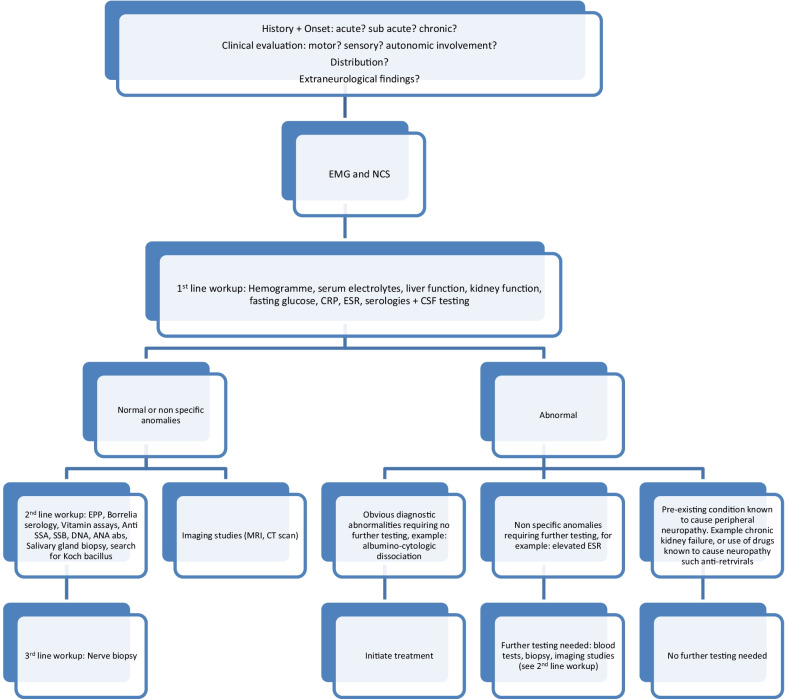
Algorithm for paraclinical investigations in patients with peripheral neuropathies. *ANA* antinuclear antibody, *CRP* c-reactive protein, *CSF* cerebrospinal fluid, *CT* computed tomography, *DNA* deoxyribonucleic acid, *EPP* electrophoresis of proteins, *EMG* electromyography, *ESR* erythrocyte sedimentation rate, *MRI* magnetic resonance imaging, *NCS* nerve conduction studies, *SSA* Sjogren syndrome-A, *SSB* Sjogren syndrome-B

The algorithm for paraclinical investigations entailed standard laboratory tests, cerebrospinal fluid (CSF) testing and electroneuromyography (ENMG). Standard laboratory tests included hemogramme, renal function (blood urea nitrogen and creatinine), hepatic function (liver transaminases, gamma-glutamyl transferase, alkaline phosphatase), C-reactive protein, erythrocyte sedimentation rate (ESR), fasting glucose, electrolytes, thyroid tests and serologies for syphilis and the human immunodeficiency virus (HIV). CSF testing entailed cytology and search for pathogens, as well as CSF protein and glucose levels. Mycobacterium species polymerase chain reaction (PCR) and search for neoplastic cells were ordered if the clinical picture was suggestive of these etiologies accordingly. Further testing was ordered based on the outcomes of the above initial testing. Patients with macrocytic anemia, for example, went on to have vitamin B12 assay ordered. Imaging studies were required for patients presenting other manifestations other than peripheral neuropathy or patients in whom the etiology required imaging to orient the diagnosis as is the case of neoplasms (see Fig. 1).

The study was reviewed and approved by the Ethics Committee of the Faculty of Medicine and Pharmacy of Marrakesh in accordance with the Declaration of Helsinki. The requirement of patient consent was waived by the ethics committee seeing as data were gathered from department archives and did not involve intimate details of any patient in particular.

## Results

The study comprised 180 patients in all. A male predominance of 68.9% and a sex ratio of 2.2 were observed.

**Fig. 2:**
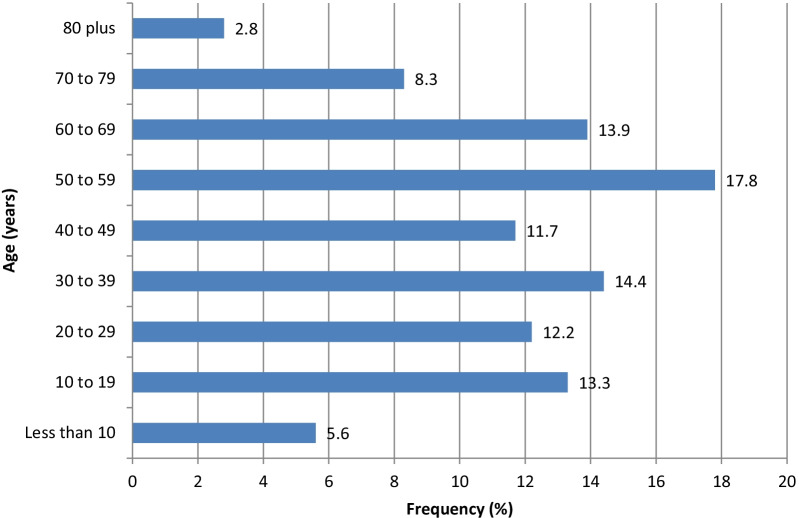
Distribution of cases per age of patient

The mean age of patients was 42.35 ± 18.76 years. A peak frequency of 17.8% was observed in the 50–59 age group. Patients aged 40 years or more accounted for over 50% of the sample (see Fig. 2).

Motor symptoms were the most frequent presentations in patients with up to 86.6% of patients reporting weakness (12.7%), cramps or fasciculations (40.5%) and peroneal atrophy (47.2%). Sensory (50.5%) and neurovegetative (38.4%) manifestations were also quite common (see Table 2).

**Table 2:** Distribution of symptoms prompting consultation by patient

	Patient complaints	*n*	Frequency (%)
Motor signs 86.6%	Weakness	23	12.7
	Crampes/ fasciculations	73	40.5
	Peronial atrophy	85	47.2
Sensory signs 50.5%	Paresthesias	60	33.3
	Dysesthesias	16	8.9
	Hypesthesia	24	13.3
	Pain	19	10.5
	Equilibrium problems	10	5.5
Neurovegetative signs 38.4%	Malaise (post prandial, orthostatic)	42	23.3
	Sweat problems	2	1.1
	Problems with micturition	35	19.4
	Sexual disorders (erection, ejaculation)	0	0
	Motility diarrhea	19	10.5
	Trophic disorders	2	1.1

Laboratory, imaging and neurophysiological investigations were ordered in the diagnostic workup of our patients. Routine labs such as hemogramme (172/180), renal (159/180) and hepatic function (127/180) and inflammation markers (155/180) were the most commonly ordered tests. Cerebrospinal fluid examination (126/180) was also a frequent test in the workup of patients. Conversely, immunological tests such as anti-SSA and anti-SSB, anti-DNA and ANA as well as vitamin assays were not frequently ordered. This was also true for neuroimaging (see Table 3).

**Table 3:** Distribution of lab workup and neuroimaging

		Ordered		Normality status	
		Not ordered	Ordered	Normal	Not normal
Hemogramme		8	172	120	52
Renal function		21	159	149	10
Hepatic function		53	127	118	9
Hepatitis serology		174	6	6	0
CRP		25	155	142	13
ESR		30	150	117	33
Glycemia		18	162	92	70
Electrolytes		87	93	84	9
CSF		54	126	79	47
EPP CSF		102	58	10	48
Thyroid function		170	10	10	0
VDRL		26	154	148	6
TPHA		26	154	147	7
HIV		70	110	108	2
EPP blood		102	58	10	48
Lipid assay		88	92	66	26
Borrelia serology		169	11	11	0
Vitamin assay	Vit B12	170	10	8	2
	Vit E	172	8	8	0
Immunological assay	Anti SSA (Ro), SSB (La)	165	15	9	6
	Anti DNA	167	13	13	0
	Latex/ Waler Ross	166	14	12	2
	ANA	170	10	10	0
Salivary gland biopsy		160	20	5	15
CPK–LDH		165	15	10	5
Koch bacillus		161	19	18	1
Porphyrine dosing		179	1	0	1
Nerve biopsy		176	4	0	4
Neuroimaging	CT scan	160	20	13	7
	MRI	154	26	16	10

ANA: antinuclear antibody, CBC: complete blood count, CPK: creatine phosphokinase, CRP: c-reactive protein, CSF: cerebrospinal fluid, CT: computed tomography, DNA: deoxyribonucleic acid, EPP: electrophoresis of proteins, ESR: erythrocyte sedimentation rate, FBG: fasting blood glucose, HIV: Human immunodeficiency virus, LDH: lactate dehydrogenase, MRI: magnetic resonance imaging, SSA: Sjogren syndrome-A, SSB: Sjogren syndrome-B, TPHA: *Treponema pallidum* hemagglutination assay, VDRL: venereal disease research laboratory.

**Fig. 3:**
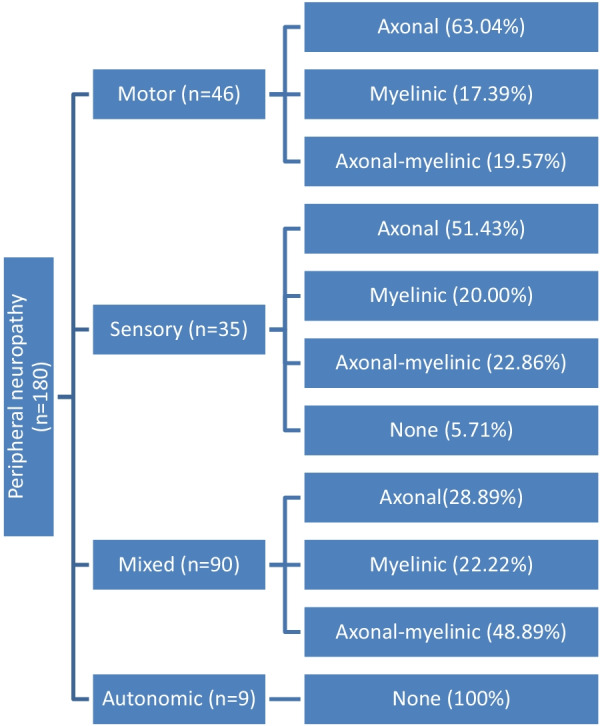
Electrophysiological distribution of motor, sensory, sensorimotor and autonomic peripheral neuropathies

Electrophysiological investigation was indicated in all our patients with the majority (50%) presenting mixed neuropathy. The axonal form (40.56%) was the most frequent (see Fig. 3).

The most common etiologies found in the present study are diabetes (26.7%), acute polyradiculoneuropathy or APRN (26.1%), and amyotrophic lateral sclerosis or ALS (16.1%). Rare causes of peripheral neuropathies include toxic (0.6%) and amyloid (0.6%) mechanisms. Only in 5% of cases was a cause not determined; see Additional file 1: Table S1.

Outcome was mostly favorable under treatment, although 10 deaths due to acute polyradiculoneuropathy were recorded.

## Discussion

The main findings in our study are the frequency of motor symptoms, the preponderance of the axonal form and the dominance of diabetes, APRN and ALS in etiological investigations. To the best of our knowledge, this is the first hospital-based study on peripheral neuropathy in southern Morocco.

One of the challenges in comparative studies on etiological profiles is that several studies are demographic- or type-specific in that they explore neuropathies in the elderly only or in diabetic patients only. There is also the problem of definition. Different studies tend to define or group diagnoses together. As an example, Hanewinckel and colleagues defined inflammatory neuropathies as including “Guillain–Barré syndrome, chronic inflammatory demyelinating polyradiculoneuropathy, and neuropathies associated with paraproteins, paraneoplastic antibodies, and infections (human immunodeficiency virus, Lyme disease, leprosy).” This led to a prevalence of 13% for inflammatory neuropathies and this was distinct from connective tissue disease/vasculitis-associated neuropathies which had a prevalence of 4% [8]. Verghese and colleagues, however, distinguished inflammatory-demyelinating, cancer, MGUS (monoclonal gammopathy of undetermined significance), vasculitis, and infection. Unlike Hanewinckel and colleagues, however, they restricted their study to the “young-old” and the “old-old” [9]. Much like Verghese and colleagues, Visser and colleagues clustered etiologies [5]. This was not the case in our study. These make it difficult to have a clear general picture of etiological profiles and the relativity that exists across geographical zones.

A finding in our study consistent with patterns in the literature is the quasi-constant preponderance of diabetes [5, 8, 9]. A population-based study conducted in Egypt placed diabetes atop the list of non-compressive neuropathies [10]. A similar trend was reported in a study in Nigeria [11]. In a Norwegian study, however, idiopathic neuropathies were the most frequent in their sample [12]. In our study, idiopathic polyneuropathies represented only 5% of the sample and occupied the fifth position together with medication-associated polyneuropathies. A general trend in the literature is the gradual dwindling of idiopathic-tagged neuropathies with the advent of better diagnostic tools and the greater recognition of hereditary and immune-mediated causes which were much more difficult to distinguish in the past [13, 14]. Indeed, it has been suggested that both cryptogenic neuropathies and diabetic neuropathy might share common risk factors and a common underlying pathology [15]. The steep difference between diabetic and idiopathic polyneuropathies in our sample attests to the extensiveness of investigations in a quest to label neuropathies. Interestingly, Rudolph and colleagues report the absence of a cause in 28% of their sample [12]. Table 4 summarizes our findings compared to other hospital-based populations.

**Table 4:** Comparison of etiologies of peripheral neuropathies with four other studies

	Our cohort (*n* = 180)	Egypt [9] (*n* = 1343)	Norway [12] (*n* = 226)	Netherlands [5] (*n* = 743)	Meta-analysis [8] (*n* = 2613)
Diabetes	26.7%	20.40%	18%	32%	34%
Inflammatory	27.2%	0.74%	16%^a^	9%^c^	13%^c^
APRN	26.1%	0.37%	12%	–	–
CPRN	1.1%	0.37%	4%	–	–
ALS	16.1%	–	–	–	–
Immunological disease	5.6%	–	4%	5%	4%
Idiopathic	5%	2.90%	28%	26%	23%
Medication	5%^f^	–	–	–	–
Infectious	3.9%	0.74%	2%	–	–
Hereditary	–	0.37%^b^	14%^b^	5%	6%
Friedreich’s disease	2.8%	0.22%	–	–	–
Alcohol	2.2%	–	10%	–	–
Paraneoplastic	1.7%	0.07%	3%	–	–
Critical illness polyneuropathy	1.7%	–	–	–	–
Deficiency neuropathy	1.1%	–	4%	4%	3%
Amyloid	0.6%	–	–	–	–
Toxic	0.6%	–	–	14%^d^	13%^d^
Metabolic	–	21.89%^e^	–	4%^e^	4%^e^
Hypoythroidism	–	0.37%	4%	–	–
Liver disease	–	0.45%	1%	–	–

^a^Inflammatory neuropathy = APRN, CPRN

^b^Hereditary causes such as Charcot-Marie-Tooth were not found in our study; for reference 9, figure represents only Charcot-Marie-Tooth

^c^Chronic inflammatory demyelinating polyneuropathy, Guillain–Barré syndrome, polyneuropathy associated with monoclonal gammopathy of undetermined significance, polyneuropathy associated with malignancy/neuronal paraneoplastic antibodies, and HIV, Lyme disease, leprosy-associated polyneuropathy

^d^Vasculitic neuropathy, amyloid neuropathy, sarcoid neuropathy, and polyneuropathy with connective tissue disease, such as Sjögren disease and rheumatoid arthritis

^e^Uremic polyneuropathy and neuropathy related to thyroid dysfunction, renal failure, and liver disease

^f^Medication-induced neuropathy in our study was due to cisplatine in 5 patients and isoniazide in 4 patients

It is crucial, when faced with a patient with peripheral neuropathy, to first exclude emergencies. Guillain–Barré syndrome (GBS) is the peripheral neuropathy emergency *par excellence*. It is the idiopathic form of the APRN. Clinically, it is an acute bilateral, symmetrical, synchronous, ascending motor and/or sensory impairment. The risk of cardiovascular dysautonomic symptoms and the involvement of chest and bulbar muscles lead to a high mortality of the condition [16]. The ten deaths recorded in the study were due to APRN alone, resulting in a mortality of 21.3% of APRN cases. This is much higher than other series in the literature [16, 17]. This might be due to delay in diagnosis and limited means for adequate management of patients in an intensive care setting. APRN was the second most frequent form of peripheral neuropathy after diabetes in the present study. Epidemiological studies place GBS much further below diabetic neuropathy in frequency, with an annual incidence of 1–2 and 23–54 per 100,000 persons, respectively [8]. There is a relatively high prevalence of APRN in our sample. This could be due to the fact that neuropathy in other diseases such diabetes and systemic illnesses tends to be managed by other specialists. APRN; however, being an emergency tends to always fall to the neurologist.

In APRN, it is understood that the peripheral nervous system is caught in the crossfire between the immune system and pathogens in the body by molecular mimicry. The role of *Campylobacter jejuni* in the pathogenesis of the aberrant immune response that leads to auto-attack by antibodies has been well documented [18]. In like manner, growing evidence points to the implication of several other pathogens as is the case in the present study [19]. Causes found included *Helicobacter pylori*, *Treponema pallidum* and *Mycobaterium tuberculosis* infections. More recently, COVID-19 infection and vaccines have also been implicated [2]. Typically, electrophysiological data point to a demyelinating process. However, geographical variations have been observed with Western countries reporting demyelinating disease, whereas Asian region reporting axonal forms [20, 21]. In addition, a chronic form of polyradiculoneuropathy (CPRN) was found to have a much lower prevalence in this study. It generally causes more morbidity than mortality as compared to APRN [22].

Peripheral neuropathy due to diabetes is the most encountered scenario in the clinical setting. However, it should be considered a diagnosis of exclusion. Its prevalence has been estimated to be between 200 and 600 cases per 100,000 [8]. The presentation could be multifaceted and require exploration of other etiologies rather than settling down for the obvious diagnosis. Typically, patients present with sensory symptoms ranging from hypoesthesia to dysesthesia. Autonomic findings are also frequent and might involve erectile issues in males along with gastroparesis or constipation and urinary difficulties. Diabetic neuropathy occurs in patients who have had a long history of diabetes and typically uncontrolled blood glucose levels. This glucose toxicity leads to axonal damage. The link between blood glucose and neuropathy seems much less straight forward in type 2 diabetes than it is in type 1 diabetes. Several risk factors such as hypertriglyceridemia, dyslipidemia, obesity and hypertension might be implicated in neuropathology, and modifying them might help keep impairment at bay [23]. Diabetic neuropathy is a risk factor for falls and amputations. In fact, diabetic neuropathy is associated with an increased risk of mortality in patients [24].

The algorithm for ordering paraclinical investigations is based on the rule of using a justifiable number of tests with the greatest diagnostic returns. It is helped by the trends in regional frequency of causes and the ease of obtaining tests to make diagnoses. This requires knowledge of regional profiles of neuropathies, and available means. In addition, it is important to take note of patient history and the clinical picture. Routine tests such as the complete blood count (CBC), erythrocyte sedimentation rate (ESR), fasting blood glucose (FBG), blood electrolytes, renal, liver and thyroid function are an initial step toward making an accurate diagnosis [25]. In addition, in our setting, it is routine practice to order syphilis serology and CSF routine tests. Vitamin B12 assay is routinely ordered according to series in the literature but this was only ordered in 10/180 cases in this series.

Also, it is important to perform electrophysiological evaluation to classify the neuropathy type. Nerve conduction studies (NCS) would define the axonal or myelinic type and the sensory or motor type. In our setting, these routine tests were the most frequently ordered in case of peripheral neuropathies.

These tests could give an idea about the underlying pathology to the neuropathy. Elevated ESR might signal an inflammatory disorder or a neoplastic origin. Diabetes could be suspected on FBG, and renal, liver or thyroid dysfunction could be diagnosed on routine tests. In case of macrocytic anemia, for example, vitamin assays should be ordered. In the present work, vitamins B12 and E were the two vitamin assays ordered. In our study, deficiency neuropathy was present in 1.1% of our patients which is far lower than a Nigerian study which reported a frequency of 10.1% [11]. Visser et al., however, did not find deficiency neuropathies in their study in a Dutch population [5]. The differences in prevalence of deficiency neuropathies might be due to differences in standards of living. Deficiency neuropathies in developed countries might be associated with alcohol abuse and the accompanying denutrition, rather than malnutrition. Vitamin deficiency neuropathies are typically sensory neuropathies [26]. The mechanisms by which this deficiency occurs are multiple and sometimes multifactorial. They are dominated by malnutrition, malabsorption syndrome, chronic pancreatitis, as well as alcohol and tobacco use because of the undernutrition they cause [27]. A related neuropathy is critical illness polyneuropathy (CIP). Denutrition has been suggested as possible cause. However, this and the implication of other factors remain speculative [28]. Deficiency neuropathies and CIP are relatively less frequent in our population sample.

An important cause of peripheral neuropathies in our patients is alcohol abuse. It is twice as frequent as deficiency neuropathies and nearly four times as common as other toxic causes put together. This is interesting given the religious context in Morocco, where alcohol is prohibited. Alcohol neuropathy is classically a sensory neuropathy due to direct toxicity of alcohol and the implication of nutritional deficiencies associated with alcohol abuse [29].

Vasculitic neuropathies are also an important etiological mechanism of peripheral neuropathies. These could be encountered in the context of systemic illnesses, infections, paraneoplastic and drug-induced disorders [30]. Extensive laboratory workup is required in such instances. It is important to test for anti-ganglioside antibodies, anti-myelin associated glycoprotein, angiotensin-converting enzyme titer, anti-nuclear antigen profile, anti-neutrophil cytoplasmic antigen antibody profile, cryoglobulins, anti-Ro, anti-La, rheumatoid factor, as well as anti-Caspr1/2, anti-LGi1 and anti-ganglionic acetylcholine receptor antibodies. In addition, infectious serologies (hepatitis B and C, HIV, syphilis) should be performed [1]. Patients in this study with systemic illness-associated neuropathy were found to have Sjogren, rheumatoid arthritis and polyarteritis nodosa. Vasculitic neuropathies are usually associated with extra-neurological symptoms and constitutional symptoms. The classic clinical presentation is painful mononeuritis multiplex [31]. Infections such hepatitis C, Human immunodeficiency virus (HIV), *Borrelia burgdorferi* and Hansen bacillus infections were incriminated in infectious neuropathies in our cohort. Drug-induced neuropathies could also be encountered in the process of treatment of these neuropathies. Anti-retrovirals and anti-tuberculosis drugs, such as isoniazid are known culprits [32]. In addition, paraneoplastic neuropathies are worth determining as this might lead to an early diagnosis of a tumor [33].

These cases require extensive workup to come up with an exact diagnosis for proper treatment. Immunological tests in CSF and serum are crucial. Radiological imaging is also necessary. For patients presenting with paraneoplastic neuropathy in the present cohort, two patients were diagnosed with kidney tumors using magnetic resonance imaging (MRI), while a third had unlabeled abdominal masses on ultrasound evaluation. In addition, nerve biopsy in challenging cases could make a difference. In our cohort, nerve biopsy was ordered only in 4 cases.

In amyloid neuropathy, nerve biopsy is essential. Salivary gland and skin biopsies could also be performed to search for abnormal beta-sheet fibrillar protein aggregates. In the present study, only one case of amyloid neuropathy was identified, a testament to the rarity of the condition in our population. This single case was the subject of a case report in 2009 [34]. Amyloid neuropathy typically involves the autonomic system. It occurs in a context of systemic involvement including cardiac hypertrophy, hepatomegaly, renal and hematological disorders [3].

ALS is an important cause of peripheral neuropathy coming third in frequency in the present study. However, it involves both the upper and lower motor neurons such that the condition is characterized clinically by an association of both central and peripheral motor impairment. No sensory involvement is associated. Patients typically present fasciculations suggestive of anterior horn cell involvement and confirmed on NCS [35]. It is important to point out the presence of primary and secondary forms. The majority of cases in this study were of primary origin with only 5 cases imputed to syphilis. HIV-associated ALS has also been reported in other series [36].

Like the above, Friedreich’s disease involves both the central and peripheral nervous systems. However, unlike ALS, its systemic involvement is far-reaching and responsible for a clinical picture including several neurological findings, such as motor and sensory neuropathies, cerebellar impairment, visual and auditory issues, as well as musculoskeletal deformities, cardiovascular problems and diabetes [4]. The condition is an autosomal recessive disorder and, given the high rate of consanguinity in several areas in the country, we suspect there might be a higher prevalence of the condition in Morocco than elsewhere [37]. Out of the 5 cases identified in our patients, 2 were a brother and a sister from a consanguineous marriage.

The main limitation of the study is that we used a hospital-based cohort which might in reality be nothing like what goes on in the general population. However, it is important to point out that such a study, especially this convenience cohort, is important in revealing hospital-relevant diagnoses. That is, we know better the etiological profile of patients whose neuropathy would tend to land them in the hospital, and more specifically, in the Neurology ward. This knowledge is more important to the hospital practitioner than a population profile per se. Despite exhaustive investigations, an etiology was not found in 5% of cases.

## Conclusions

Peripheral neuropathies are quite common in clinical practice and can constitute a diagnostic challenge for physicians in terms of their various etiologies. Though no exact tally exists between clinical presentation and etiology, a thorough semiological analysis together with electrodiagnostic data and routine lab tests could be of great value in arriving at the right diagnosis. In case of an acute presentation, it is important to exclude emergencies such as GBS which could be life-threatening. The presence of extra-neurological signs such as vasculitic skin lesions, arthralgia and constitutional symptoms could also have an important orienting value. Sometimes, extra-neurological involvement could be a determining factor in prognosis. These ‘neurological plus’ conditions might be revealed by neuropathies, hence making the case for the need to arrive at the right diagnosis. Knowledge of regional profiles comes in to enhance chances of making the right diagnosis.

## Supplementary Information

**Additional file 1**: **Table S1**. Electrophysiological distribution of various peripheral neuropathy etiologies.

## Abbreviations

ALS: Amyotrophic lateral sclerosis
ANA: Antinuclear antibody
APRN: Acute polyradiculoneuropathy
Caspr: Contactin-associated protein
CBC: Complete blood count
CIP: Critical illness polyneuropathy
CPK: Creatine phosphokinase
CPRN: Chronic polyradiculoneuropathy
CRP: C-reactive protein
CSF: Cerebrospinal fluid
CT: Computed tomography
DNA: Deoxyribonucleic acid
ENMG: Electroneuromyography
EPP: Electrophoresis of proteins
ESR: Erythrocyte sedimentation rate
FBG: Fasting blood glucose
GBS: Guillain–Barré syndrome
HIV: Human immunodeficiency virus
LDH: Lactate dehydrogenase
LGI: Leucine-rich glioma-inactivated
MGUS: Monoclonal gammopathy of undetermined significance
MRI: Magnetic resonance imaging
NCS: Nerve conduction studies
PCR: Polymerase chain reaction
SSA: Sjogren syndrome-A
SSB: Sjogren syndrome-B
TPHA: *Treponema pallidum* Hemagglutination assay
VDRL: Venereal disease research laboratory

## Declarations

### Availability of data and materials

Data obtained in the present study are freely available and could be obtained on written request to the corresponding author.
