# Supplementary material for: Etiological profile of peripheral neuropathies in an academic hospital in southern Morocco
Source: Egypt J Neurol Psychiatr Neurosurg. 2022 Aug 20;58(1):97. doi: 10.1186/s41983-022-00531-4 (PMC9391624; doi:10.1186/s41983-022-00531-4)
Supplement: Supplementary file 1 — Additional file 1: Table S1. Electrophysiological distribution of various peripheral neuropathy etiologies. [file 41983_2022_531_MOESM1_ESM.docx]

Table S1: Electrophysiological distribution of various peripheral neuropathy etiologies

|  | **Neurophysiological results** | | | |
| --- | --- | --- | --- | --- |
| **Etiology** | **Motor** | **Sensory** | **Mixed** | **Autonomic** |
| Diabetes (n=48) | 29.17% | 10.42% | 56.25% | 4.17% |
| Acute polyradiculoneuropathy (n=47) | 4.26% | 10.64% | 85.11% | 0% |
| Motor neuron disease (n=29) | 100% | 0% | 0% | 0% |
| Immunologic (n=10) | 0% | 50% | 0% | 50% |
| Idiopathic (n=9) | 0% | 77.78% | 22.22% | 0% |
| Medication (n=9) | 0% | 0% | 100% | 0% |
| Infectious (n=7) | 0% | 0% | 100% | 0% |
| Friedreich’s ataxia (n=5) | 0% | 100% | 0% | 0% |
| Alcohol (n=4) | 0% | 100% | 0% | 0% |
| Paraneoplastic (n=3) | 33.33% | 33.33% | 0% | 33.33% |
| Critical illness polyneuropathy (n=3) | 0% | 0% | 100% | 0% |
| Chronic polyradiculoneuropathy (n=2) | 0% | 0% | 100% | 0% |
| Deficiency neuropathy (n=2) | 0% | 100% | 0% | 0% |
| Amyloid (n=1) | 0% | 0% | 0% | 100% |
| Toxic (n=1) | 0% | 100% | 0% | 0% |
